# Low Dose Betahistine in Combination With Selegiline Increases Cochlear Blood Flow in Guinea Pigs

**DOI:** 10.1177/00034894221098803

**Published:** 2022-06-03

**Authors:** Benedikt Kloos, Mattis Bertlich, Jennifer L. Spiegel, Saskia Freytag, Susanne K. Lauer, Martin Canis, Bernhard G. Weiss, Friedrich Ihler

**Affiliations:** 1 Department of Otorhinolaryngology, University Hospital, LMU Munich, Munich, Germany; 2 Institute of Surgical Research, Walter-Brendel-Centre of Experimental Medicine, University Hospital, LMU Munich, Munich, Germany; 3 German Center for Vertigo and Dizziness (DSGZ), LMU Munich, Munich, Germany; 4 Department of Dermatology and Allergy, University Hospital, LMU Munich, Munich, Germany; 5 Molecular Medicine, Harry Perkins Institute of Medical Research, Perth, WA, Australia; 6 Clinic for Small Animal Surgery and Reproduction, LMU Munich, Munich, Germany; 7 Department of Ear, Nose and Throat Diseases, Head and Neck Surgery, Greifswald, Germany

**Keywords:** betahistine, selegiline, cochlea, microcirculation, guinea pig, Menière’s disease

## Abstract

**Objective:**

Betahistine is frequently used in the pharmacotherapy for Menière’s Disease (MD). Little is known about its mode of action and prescribed dosages vary. While betahistine had an increasing effect on cochlear microcirculation in earlier studies, low dose betahistine of 0.01 mg/kg bw or less was not able to effect this. Selegiline inhibits monoaminooxidase B and therefore potentially the breakdown of betahistine. The goal of this study was to examine whether the addition of selegiline to low dose betahistine leads to increased cochlear blood flow.

**Methods:**

Twelve Dunkin-Hartley guinea pigs were anesthetized, the cochlea was exposed and a window opened to the stria vascularis. Blood plasma was visualized by injecting fluoresceinisothiocyanate-dextrane and vessel diameter and erythrocyte velocity were evaluated over 20 minutes. One group received low dose betahistine (0.01 mg/kg bw) and selegiline (1 mg/kg bw) i.v. while the other group received only selegiline (1 mg/kg bw) and saline (0.9% NaCl) as placebo i.v.

**Results:**

Cochlear microcirculation increased significantly (*P* < .001) in guinea pigs treated with low dose betahistine combined with selegiline by up to 58.3 ± 38.7% above baseline over a period of up to 11 minutes. In one guinea pig, the increase was 104.6%. Treatment with Selegiline alone did not affect microcirculation significantly.

**Conclusions:**

Low dose betahistine increased cochlear microcirculation significantly when combined with selegiline. This should be investigated in further studies regarding dose-effect relation in comparison to betahistine alone. Side effects, in particular regarding circulation, should be considered carefully in view of the clinical applicability of a combination therapy in patients with MD.

## Introduction

Menière’s Disease (MD) is a condition associated with frequent vertigo attacks, tinnitus and short-term sensorineural hearing loss.^1^ Prevalence is higher in female patients (64.5%) with an overall incidence rate of 13.1 per 100 000 person-years (in the United Kingdom).^2^ Menière’s Disease varies in its manifestations and there are possibly several subgroups, each resulting from a different pathophysiology.^3,4^ The most common theory is that endolymphatic hydrops in the labyrinth leads to increased inner ear pressure.^5,6^ It is believed that increased pressure in the cochlea results in mechanical strain on membranous tissues. Consequently, barriers of the endolymphatic space dilate and eventually rupture. This causes symptoms and histological abnormalities observed in patients with MD.^7-10^ Menière’s Disease is a chronic condition without causal treatment to date. Betahistine, a medication with reportedly safe drug profile has become the first-line treatment for patients with MD.^11^ Alternative non-destructive treatment options like intratympanic steroid injection or endolymphatic sac decompression surgery are more invasive and accompanied with potential complications.^1,12^

Betahistine is a histamine-like drug that binds mainly to H_3_ receptors.^13,14^ Betahistine binding to H_3_ receptors in the inner ear is supposed to increase local blood flow via dilation of the precapillary arterioles which results in increased fluid exchange. Enhanced blood flow increases reabsorption of endolymph and decreases inner ear pressure.^15-17^ Betahistine has long been an essential part of the treatment of MD ever since 1968.^18^ While definite proof of a therapeutic effect of betahistine in MD has yet to be provided,^19,20^ it has been used for over 50 years and many studies indicate a better quality of life and less frequent attacks.^11,21,22^

However, betahistine has a high first-pass effect, is rapidly broken down and has a short half-life of only up to 3.5 hours in humans.^18^ The enzyme monoaminoxidase B (MAO-B) converts betahistine into aminoethylpyridine, hydroxyethylpyridine and mainly 2-pyridylacetic acid (2-PAA).^23^ The effect of 2-PAA, however, differs in comparison with unmetabolized betahistine on cochlear microcirculation. In a study by Bertlich et al,^16^ 2-PAA proved non-effective in increasing cochlear blood flow compared to its precursor. A way to inhibit the breakdown of betahistine through MAO-B may increase betahistine levels in cochlear microcirculation.

Selegiline is a potent, dose-dependently selective and irreversible MAO-B inhibitor^24^ and has shown no major side effects in clinical use.^25^ For now, most studies on selegiline focus on its effects in the central nervous system.^26,27^ Selegiline is already available and in therapeutic use, since MAO-B activity has an influence on illnesses like Parkinson’s disease.^24^ In a cat model, oral medication with betahistine in combination with selegiline obtains therapeutic effects similar to betahistine in higher dosage.^28^

The goal of this study was to evaluate the added effect of MAO-B-inhibition by selegiline in combination with betahistine on cochlear blood flow.

## Materials and Methods

Experiments were performed according to state and animal protection law (Regierung von Oberbayern, Munich, Germany; license no. ROB-55.2-2532.Vet_02-17-231). Twelve female guinea pigs (Dunkin-Hartley) of 8 to 12 weeks of age were acquired from Envigo Laboratories (Venray, The Netherlands) and acclimated for 1 week prior to the experiment. The animal model used in this experiment has been applied extensively and has proven reliable.^14,15,29-32^

**Figure 1.**
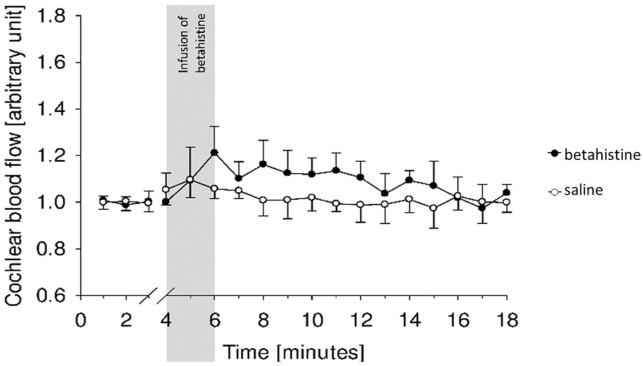
Betahistine effect on cochlear microcirculation by Ihler et al: Betahistine (0.01 mg/kg bw) alone was not able to increase cochlear blood flow significantly. Image extracted from Ihler et al.^15^

Each guinea pig was randomly assigned to intravenous treatment with betahistine combined with selegiline (Group *B* + S, n = 6) or selegiline combined with 0.9% saline solution (Group *S* + S, n = 6). Results with solely betahistine without adding selegiline have already been obtained by this group (Figure 1).^15^ The present experiments were designed in a similar way in accordance with the principles of Russel and Burch to reduce the use of animals in testing.^33^

Anesthesia was induced by intramuscular injection of 1.0 mg/kg bw midazolam (Dormicum^®^, Hoffmann-La Roche AG, Basel, Suisse), 0.2 mg/kg bw medetomidine (Domitor^®^, Vétoquinol, Magny-Vernois, France) and 0.025 µg/kg bw fentanyl (Fentadon^®^, Dechra Pharmaceuticals PLC, Northwich, UK). Depth of anesthesia was controlled by toe-pinching reflexes. Anesthesia was considered sufficiently deep with lack of these reflexes. Reflexes were monitored and one-third of the initial anesthesia dosage was injected intramuscularly if reflexes were positive.

**Figure 2.**
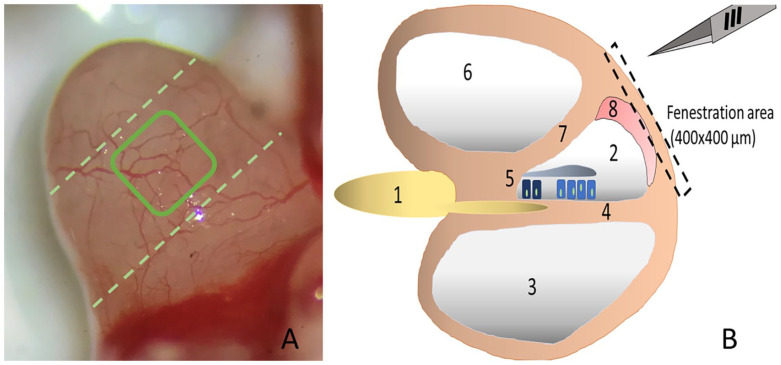
Cochlear fenestration: Image (A) shows the protruding left cochlea of a guinea pig into the tympanic cavity (3.2× magnification) before the outer vessels are removed with a microsponge (lateral view). The rectangle indicates the window which is carved into the outer bony layer of the cochlea wall. The second turn of the cochlea is marked by broken lines. Image (B) outlines the area of fenestration. The cochlea is innervated by the cochlear nerve (1), whose fibers run in between the scala media (2) and scala tympani (3) along the basilar membrane (4) to the organ of Corti (5) with inner and outer hair cells and the tectorial membrane. Between the scala vestibuli (6) and the scala media is Reissner’s membrane (7). The stria vascularis (8) lateral to the scala media is accessed by carefully removing the upper bony layer of the cochlear wall.

Jugular and periaural regions were infiltrated subcutaneously with bupivacaine (0.5 ml) with epinephrine (Bupivacain 0,5%-ig^®^, Jenapharm, Jena, Germany). An intravenous catheter (Portex^®^, 0.58 mm, Smiths Medical, St. Paul, MN, USA) was placed in the external jugular vein. The pinna, surrounding tissue and the muscles covering the temporal bone were resected en bloc. After incising the bony part of the external auditory canal with pliers (Scholl^®^, SSL International, London, UK), a lateral bulla osteotomy was performed removing the lateral wall with forceps. The cochlea was exposed with forceps after the ear drum and the ossicles were removed. Vessels covering the cochlea were carefully wiped off with a microsponge. Then a window of approximately 400 × 400 µm was carefully carved with a scalpel (Feather disposable scalpel no. 11, Feather Safety Razor Co., Ôsaka, Japan) into the outer bony layer of the second turn of the cochlea, avoiding damage to the stria vascularis underneath (Figure 2).

Fluoresceinisothiocyanate-dextrane (FITC-Dextran, Molecular weight of 500 000, Sigma-Aldrich, Deisenhofen, Germany) was applied to visualize blood plasma in contrast to erythrocytes passing through vessels. FITC-Dextran was injected intravenously via the jugular catheter as a solution of 5% dissolved in NaCl (volume: 0.01 ml/kg bw). If necessary, the injection of FITC was repeated to intensify the contrast. Cochlear microcirculation was recorded with a Leica M205FA binocular microscope with Leica EL 6000 light source, a Leica DFC295 digital color camera attached and Leica Application Suite (LAS) software (version 3.1.2., Leica Microsystems GmbH, Wetzlar, Germany) installed. The obtained videos were analyzed using CapImage software (version 8.6.3, Dr. Zeintl Engineering, Heidelberg, Germany). This software was designed for the quantification of microcirculation^34^ and calibrated to adjust for erythrocyte velocity and capillary diameter. The path of erythrocytes can be followed with the software by tracing their position in a vessel over time. Thereby, the speed of blood flow can be calculated. Vessel diameter can be recorded by manually measuring the distance between vessel walls.

After surgical preparation, 3 representative vessels were randomly selected to record capillary diameter and blood velocity. Base values were recorded before treatment by recording for 2 minutes prior to intravenous drug administration and calculating mean blood flow. This was used as a reference value of 1.0 arbitrary units for further analyses. Guinea pigs in group *B* + S were treated once with betahistine (0.01 mg/kg bw) in combination with selegiline (1 mg/kg bw). Guinea pigs in group *S* + S were treated once with selegiline (1 mg/kg bw). Saline (0.9% NaCl) was added to match the injected volume of the *B* + S group. The betahistine dosage was chosen to match the highest betahistine dosage from previous experiments without significant effect on cochlear microcirculation.^15^ The selegiline dosage was based on previous experiments with betahistine and selegiline in cats by Tighilet et al.^28^

Capillary diameter (*d*) and intravascular blood velocity (*v*) were quantified every minute for 2 minutes before and 19 minutes after drug administration. Each minute value was measured thrice and the average of the 3 measurements was calculated. After data was recorded, guinea pigs were euthanized under deep anesthesia by cervical dislocation. Cochlear blood flow (*q*) was calculated with the formula specifically proposed for this purpose by Baker and Wayland^35^:

$$q=\left(\frac{v}{1.6}\right)*{\left(\frac{d}{2}\right)}^{2}*\pi$$

Mainly due to varying diameters of surgically accessible vessels, considerable interindividual differences exist between absolute baseline values of cochlear blood flow. To account for this, data was transformed to arbitrary units, calculated as changes from the respective baseline.

Statistical differences were assessed for each timepoint between treatment groups. Therefore, the following mixed effects model was fitted with an interaction effect for timepoint and treatment and a random effect for the animal and a nested random effect for the capillary location,

$$y_{ijt}=\alpha +\beta_{j}+B_{i}+C_{k}|B_{i}+\gamma *t+\delta *t^{2}+\rho_{j}*t+\varepsilon_{ijt}$$

Here, $y_{ijt}$ represents the percentage change of cochlear blood flow over the basal measurement, α is the intercept, $\beta_{j}$ with j=1,…,3 the treatment effect (reference group is placebo/placebo), γ is the linear effect of the time point, δ is the linear effect of the squared time point, $\rho_{j}$ the interaction effect for treatment and timepoint and $\varepsilon_{ijt}$ the random error. Time was fitted linearly and polynomial to best account for its effect. The random effect $B_{i}$ is fitted to account for an animal effect and the nested random effect $B_{i}|C_{k}$ is fitted to account for the capillary location. Random effects are fitted in order to account for unobserved heterogeneity. The model was fitted using the *lmer* function from the *lme4* package of R version 4.0.5, which relies on a restricted maximum likelihood fitting. The mixed model was applied to test for a global effect within the data. The significance was assessed using Satterthwaite’s *t*-test (as implemented in the *lmer*Test package) and a significance threshold of 0.05. Normality of residuals was assessed by inspection of QQ and partial residual plots. Individual time points were tested using a simple linear mixed model with only a fixed effect for the treatment and a random effect for the animal. Significance was again assessed by a Satterthwaithe’s *t*-test. For absolute values we used the simple linear mixed models with a fixed effect and a random effect for animal to evaluate whether there was a significant effect over baseline for each individual time point. Again, the Satterthwaithe’s *t*-test was used for assessing significance.

## Results

Already at baseline, one animal from the *S* + S group showed values that were more than fivefold increased over all other individuals. This was attributed to a technical error and all values from this animal were removed from subsequent analyses. Average cochlear blood flow in all other animals (n = 11) was 66.3 ± 23.1 µm^3^/s and 65.6 ± 26.9 µm^3^/s for the first and second minute of baseline measurements, respectively. Within treatment groups, the values were 77.6 ± 21.8 µm^3^/s and 77.1 ± 29.0 µm^3^/s for the group *B* + S (n = 6) as well as 52.7 ± 16.2 µm^3^/s and 51.8 ± 15.3 µm^3^/s for the group *S* + S ( = 5). There were no statistically significant differences between groups for baseline values.

**Figure 3.**
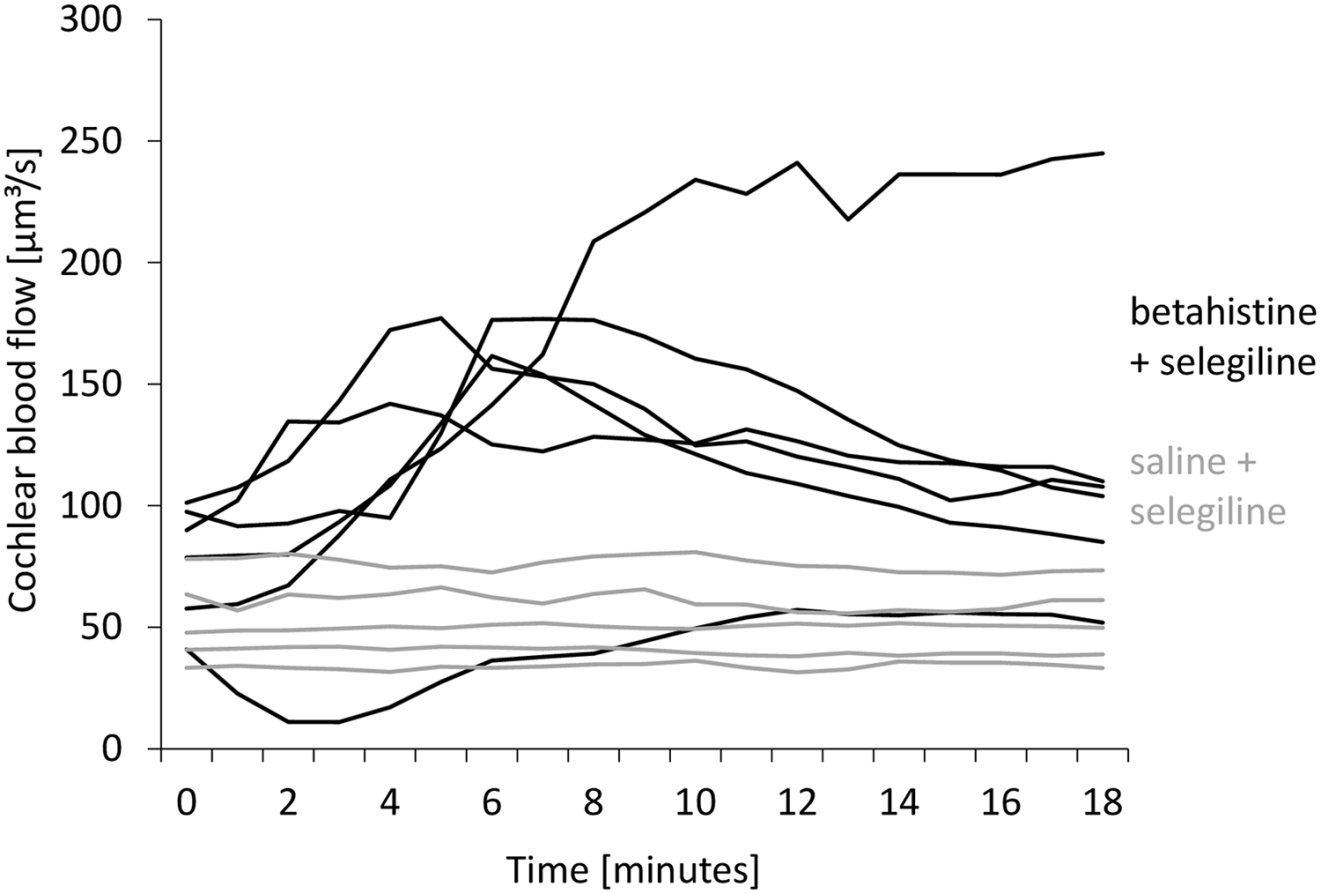
Cochlear blood flow after baseline recordings in absolute values in individual animals from the groups *B* + S (black lines, n = 6, treatment) and *S* + S (gray lines, n = 5, control). While most animals treated in *B* + S showed a marked increase in cochlear blood flow, values in the whole *S* + S group stayed within a small range.

After baseline measurements, treatment was administered once according to treatment group. Following that, maximum values ranged from 36.2 to 80.9 µm^3^/s in the *S* + S group between minutes 3 and 20. A considerable interindividual variability in absolute values of blood flow was noted within the *B* + S group: in one animal, values dropped as low as 11.0 µm^3^ in minute 3 and thereafter showed values no higher than 57.1 µm^3^ in minute 12. Four animals peaked at 141.9 to 177.2 µm^3^ in minutes 4 to 7. The last animal from *B* + S showed a consistent increase beyond minute 8, peaking at 245.0 µm^3^ at minute 18 and remaining at this level. The time course of cochlear blood flow in absolute values in individual animals is shown in Figure 3.

**Figure 4.**
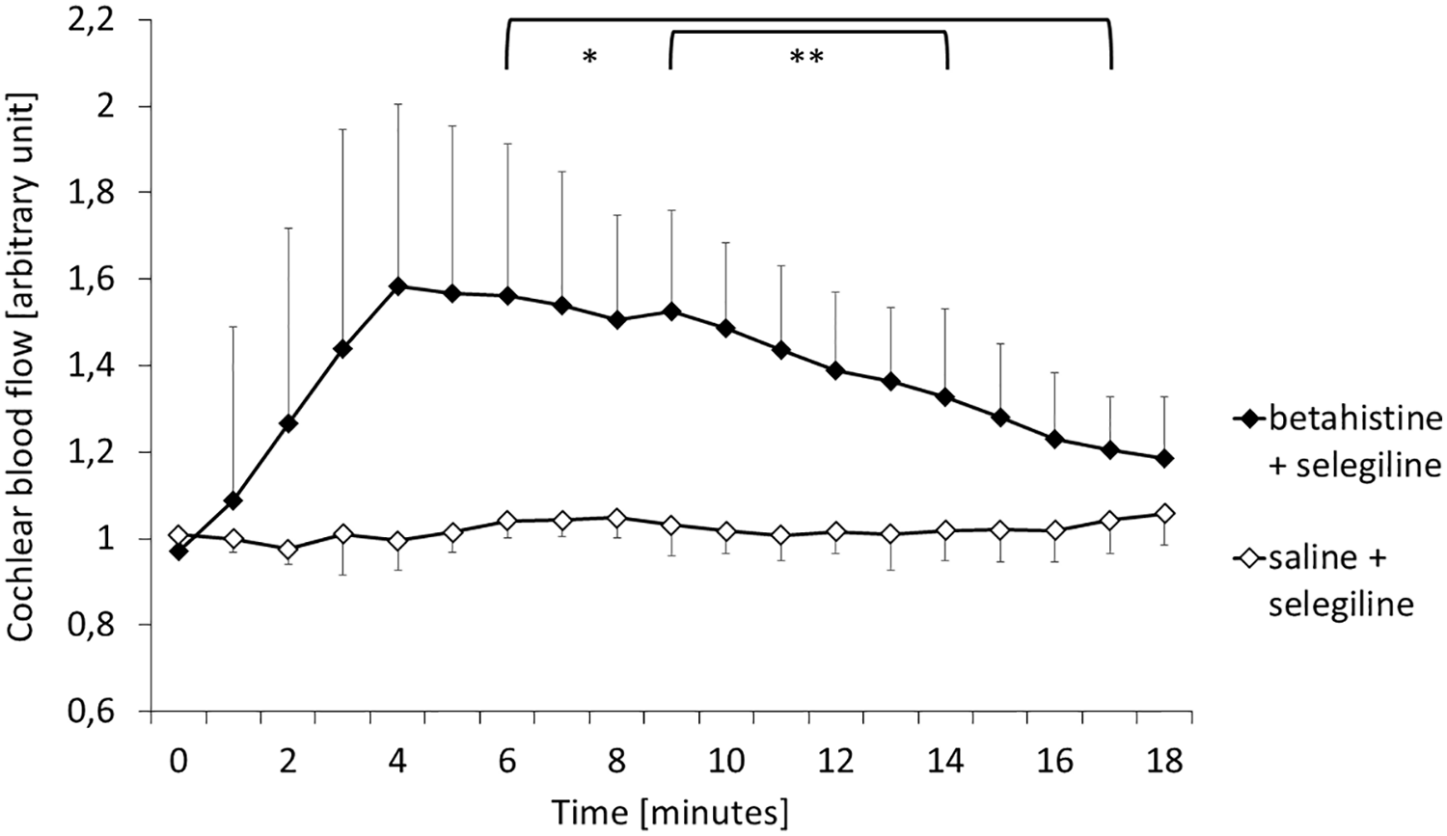
Relative change of cochlear blood flow after baseline recordings on average in both groups. Mean values ± standard deviation. Brackets: significant difference at respective timepoints between groups, level of significance denoted by asterisk (** = P < .01, * = P < .05). Group *B* + S (black diamonds, treatment, n = 6) showed a significant increase in cochlear blood flow compared to group *S* + S (white diamonds, control, n = 5).

To account for interindividual variability in absolute values irrespective of treatment group, data was calculated as change from baseline for statistical testing of differences. Thereby, cochlear blood flow after drug administration increased by a mean of 58.3 ± 38.7% in animals from group *B* + S with a maximum increase in one animal of 104.6%. Animals in group *S* + S saw changes of cochlear blood flow of 4.8 ± 7.2% with a maximum increase of 18.9% in one guinea pig. Figure 4 gives changes of cochlear blood flow within 19 minutes after treatment.

A time effect for cochlear blood flow in the *B* + S group was identified as nonlinear. Fitting of a mixed model revealed an overall significant difference between groups (*P* < .001). Furthermore, there was a significant effect for the timepoint as well as the timepoint squared. Consecutively, the difference was identified as significant from minute 6 to minute 17 (*P* < .05), as well as highly significant from minute 9 to minute 14 (*P* < .01). There were no significant interaction effects. This concludes a significant increase over a time frame of 11 minutes.

## Discussion

We were able to show that an intravenously injected combination of low dose betahistine and selegiline significantly increases cochlear microcirculation. In contrast, selegiline in combination with saline was not able to raise microcirculation.

In previous experiments, the betahistine dosage of 0.01 mg/kg bw did not increase cochlear microcirculation significantly.^15^ The design of the present study implied that as given and thereby an additional control group with betahistine of 0.01 mg/kg bw alone was not included. This, however, limits the conclusions to be drawn from the data presented here. Therefore, follow-up studies should explore further the dose-effect relations of selegilin, betahistin and a combination of those agents in direct comparison.

A possible mechanism that might explain our observation for betahistine in combination with selegiline is the inhibition of the breakdown of betahistine by MAO-B. Potentially, other animals as well as human patients may show a similar effect. An experimental study by Tighilet et al^28^ simulating a vestibular attack in cats showed a significant decrease of the plasma concentration of betahistine metabolites when combined with selegiline, specifically 2-PAA. Cats treated orally with either high dose betahistine (2 mg/kg bw) or betahistine combined with selegiline (0.2 and 1 mg/kg bw) regained normal posture significantly faster than cats treated with low dose betahistine (0.2 mg/kg bw). However, the effect on cochlear blood flow was not investigated in the study of Tighilet et al. Our results corroborate these findings.

Higher plasma levels of betahistine and its active metabolites increase blood flow to the stria vascularis of the cochlea.^15,16^ The stria vascularis is a vessel complex responsible for homeostasis within and energy supply to the inner ear. This subsequently affects hearing function.^36^ The mechanical stress caused by increased endolymphatic pressure in patients with MD may also compress vessels of the stria vascularis. The resultant reduction in oxygen supply to the inner hair cells may help explain the hearing loss in episodes of active MD.^37^ Increasing blood flow to the stria vascularis has the potential to increase partial oxygen pressure in the effected tissues.^38^ Combining selegiline with betahistine may therefore contribute to convalescence of inner hair cells after MD attacks.

By prolonging and increasing the effect of betahistine through inhibition of MAO-B, it is also notable that the dosage of betahistine required for a given effect on cochlear blood flow is reduced. Betahistine is known to demonstrate a safe drug profile with only minor side effects. However, Adrion et al^20^ conducted a study where the majority (>85%) of patients had at least one of the following adverse effects: headache, balance disorder, nausea, nasopharyngitis, feeling hot, eye irritation, and palpitations. By limiting the required dose of betahistine, systemic side effects could potentially be limited as well. However, a reduced threshold for primary effects may reduce the effect for side effects as well. Additionally, MAO-B-inhibition itself may cause other unintended effects. Blood pressure dysregulation and transient hypertensive episodes^39^ are of particular interest, since this might explain the variability noted in the animals treated with selegiline in the present study. Therefore, side-effects should be considered carefully for the potential introduction of a combination therapy of selegiline and betahistine into clinical practice.

Another benefit of selegiline in MAO-B inhibition is the decrease of reactive oxygen radicals that result from the breakdown of betahistine. The process of oxidation of the betahistine molecule produces hydrogen peroxide.^40^ This increases the oxidative stress in cells and may be toxic to its proteins.^41^ Therefore, adjunct therapy with a MAO inhibitor like selegiline may have additional therapeutic benefits, as cochlear tissue might be protected. A study by Abdanipour et al^42^ demonstrated the protective effects of selegiline in nervous tissue in vitro. Their experiment showed a significant decrease in apoptosis and necrosis in cells under oxidative stress, when pretreated with selegiline. Concurrently, Cui et al^43^ were able to show a similar effect in epithelial cells of the lung in vitro. There, selegiline decreased MAO-B activity, as well as levels of nuclear factor κB and inflammatory proteins (heme oxygenase 1 and NAD(P)H quinone dehydrogenase 1). With these findings, tissue in the cochlea may profit in a similar way.

A limitation of this study is the fact that betahistine and selegiline were administered intravenously which differs from most MD therapy protocols.^44,45^ Intravenous betahistine without the high first-pass effect of oral intake may differ in effect from the therapeutic model. However, oral medication was not possible in the animal model used here due to the invasive nature of the experiment and the need for anesthesia. Adjunct medication with selegiline inhibits conversion of betahistine into 2-PAA. Since plasma levels of unmetabolized betahistine after oral intake have been shown to be higher with adjunct oral selegiline medication,^28^ we conclude that the results of this experiment should produce similar results with oral medication.

Like selegiline, rasagiline is frequently used in Parkinson treatment. Marconi and Zwingers evaluated Unified Parkinson’s Disease Rating Scale in a comparative meta-analysis and found no significant difference between the 2 drugs.^46^ Cereda et al^47^ came to the same conclusion in a study over 3 years. This suggests that rasagiline may also be a potential addition to betahistine in MD treatment. Müller et al^48^ described a decrease of adverse effects when rasagiline instead of selegiline was used. A study to compare selegiline and rasagiline in cochlear microcirculation may determine which drug could be more beneficial.

## Conclusion

The addition of selegiline to a low dose of betahistine leads to a significant increase of cochlear microcirculation in the stria vascularis in guinea pigs. Further animal studies should explore the detailed mechanism and dose-effect relationships. Following that, the clinical safety and benefit of a combined administration to MD patients may be explored.

## References

[1] Nakashima T Pyykkö I Arroll MA , et al. Meniere’s disease. Nat Rev Dis Primers. 2016;2(1):16028. doi:10.1038/nrdp.2016.28 27170253

[2] Bruderer SG Bodmer D Stohler NA Jick SS Meier CR . Population-based study on the epidemiology of Ménière’s disease. Audiol Neurotol. 2017;22(2):74-82. doi:10.1159/000475875 28723686

[3] Ferrara S Dispenza F . Cause, pathogenesis, clinical manifestations and treatment of meniere’s disease and endolymphatic hydrops. In: Dispenza MF (ed.) Sensorineural Hearing Loss: Pathophysiology, Diagnosis and Treatment. Nova Science Publishers, Inc; 2019; 217–231.

[4] Frejo L Soto-Varela A Santos-Perez S , et al. Clinical subgroups in bilateral Meniere Disease. Front Neurol. 2016;7:182. doi:10.3389/fneur.2016.00182 27822199 PMC5075646

[5] Perez-Carpena P Lopez-Escamez JA . Current understanding and clinical management of Meniere’s disease: a systematic review. Semin Neurol. 2020;40:138-150. 31887752 10.1055/s-0039-3402065

[6] Brown DJ Chihara Y Curthoys IS Wang Y Bos M . Changes in cochlear function during acute endolymphatic hydrops development in guinea pigs. Hear Res. 2013;296:96-106. 23270618 10.1016/j.heares.2012.12.004

[7] Salt AN Plontke SK . Endolymphatic hydrops: pathophysiology and experimental models. Otolaryngol Clin North Am. 2010;43(5):971-983. 20713237 10.1016/j.otc.2010.05.007 PMC2923478

[8] Böhmer A Dillier N . Experimental endolymphatic hydrops: are cochlear and vestibular symptoms caused by increased endolymphatic pressure? Ann Otol Rhinol Laryngol. 1990;99(6):470-476. 2350132 10.1177/000348949009900611

[9] Gluth MB . On the relationship between Menière’s disease and endolymphatic hydrops. Otol Neurotol. 2020;41(2):242-249. 31746815 10.1097/MAO.0000000000002502

[10] Pender DJ . Endolymphatic hydrops and Ménière’s disease: a lesion meta-analysis. J Laryngol Otol. 2014;128(10):859-865. 25236508 10.1017/S0022215114001972

[11] Seyed Tootoonchi SJ Ghiasi S Shadara P Samani SM Fouladi DF . Hearing function after betahistine therapy in patients with Ménière’s disease. Braz J Otorhinolaryngol. 2016;82(5):500-506. 26810620 10.1016/j.bjorl.2015.08.021 PMC9444678

[12] Pullens B van Benthem PP . Intratympanic gentamicin for Ménière’s disease or syndrome. Cochrane Database Syst Rev. 2011;3:CD008234. 10.1002/14651858.CD008234.pub2 PMC13378876 21412917

[13] Lacour M Sterkers O . Histamine and betahistine in the treatment of vertigo: elucidation of mechanisms of action. CNS Drugs. 2001;15(11):853-870. 11700150 10.2165/00023210-200115110-00004

[14] Bertlich M Ihler F Freytag S Weiss BG Strupp M Canis M . Histaminergic H3-heteroreceptors as a potential mediator of betahistine-induced increase in cochlear blood flow. Audiol Neurotol. 2015;20(5):283-293. doi:10.1159/000368293 26139562

[15] Ihler F Bertlich M Sharaf K Strieth S Strupp M Canis M . Betahistine exerts a dose-dependent effect on cochlear stria vascularis blood flow in guinea pigs in vivo. PLoS One. 2012;7(6):e39086. doi:10.1371/journal.pone.0039086 22745706 PMC3380058

[16] Bertlich M Ihler F Sharaf K Weiss BG Strupp M Canis M . Betahistine metabolites, aminoethylpyridine, and hydroxyethylpyridine increase cochlear blood flow in guinea pigs in vivo. Int J Audiol. 2014;53(10):753-759. doi:10.3109/14992027.2014.917208 25014609

[17] Bertlich M Ihler F Weiss BG , et al. Role of capillary pericytes and precapillary arterioles in the vascular mechanism of betahistine in a guinea pig inner ear model. Life Sci. 2017;187:17-21. doi:10.1016/j.lfs.2017.08.015 28818391

[18] Jeck-Thole S Wagner W . Betahistine. Drug Safety. 2006;29(11):1049-1059. doi:10.2165/00002018-200629110-00004 17061910

[19] James AL Burton MJ . Betahistine for Menière’s disease or syndrome. Cochrane Database Syst Rev. 2001;1:CD001873. 10.1002/14651858.CD001873 PMC6769057 11279734

[20] Adrion C Fischer CS Wagner J Gürkov R Mansmann U Strupp M . Efficacy and safety of betahistine treatment in patients with Meniere’s disease: primary results of a long term, multicentre, double blind, randomised, placebo controlled, dose defining trial (BEMED trial). BMJ. 2016;352:h6816. doi:10.1136/bmj.h6816 26797774 PMC4721211

[21] Nauta JJ . Meta-analysis of clinical studies with betahistine in Ménière’s disease and vestibular vertigo. Eur Arch Otorhinolaryngol. 2014;271(5):887-897. 23778722 10.1007/s00405-013-2596-8

[22] Redon C Lopez C Bernard-Demanze L , et al. Betahistine treatment improves the recovery of static symptoms in patients with unilateral vestibular loss. J Clin Pharmacol. 2011;51(4):538-548. 20940335 10.1177/0091270010369241

[23] Strupp M Kraus L Schautzer F Rujescu D . Menière’s disease: combined pharmacotherapy with betahistine and the MAO-B inhibitor selegiline - an observational study. J Neurol. 2018;265(Suppl 1):80-85. doi:10.1007/s00415-018-8809-8 29532287

[24] Magyar K . The pharmacology of selegiline. In: Youdim MBH Douce P (eds) International Review of Neurobiology. Academic Press; 2011;65-84. 10.1016/B978-0-12-386467-3.00004-2 21971003

[25] Youdim MB Riederer PF . A review of the mechanisms and role of monoamine oxidase inhibitors in Parkinson’s disease. Neurology. 2004;63(7 suppl 2):S32-S35. doi:10.1212/WNL.63.7_suppl_2.S32 15477584

[26] Amini-Khoei H Saghaei E Mobini G-R , et al. Possible involvement of PI3K/AKT/mTOR signaling pathway in the protective effect of selegiline (deprenyl) against memory impairment following ischemia reperfusion in rat. Neuropeptides. 2019;77:101942. 10.1016/j.npep.2019.101942 31272684

[27] Ishikawa T Okano M Minami A , et al. Selegiline ameliorates depression-like behaviors in rodents and modulates hippocampal dopaminergic transmission and synaptic plasticity. Behav Brain Res. 2019;359:353-361. 30359642 10.1016/j.bbr.2018.10.032

[28] Tighilet B Léonard J Watabe I Bernard-Demanze L Lacour M . Betahistine treatment in a cat model of vestibular pathology: Pharmacokinetic and pharmacodynamic approaches. Front Neurol. 2018;9:431. doi:10.3389/fneur.2018.00431 29942281 PMC6005348

[29] Arpornchayanon W Canis M Ihler F Settevendemie C Strieth S . TNF-α inhibition using etanercept prevents noise-induced hearing loss by improvement of cochlear blood flow in vivo. Int J Audiol. 2013;52(8):545-552. doi:10.3109/14992027.2013.790564 23786392

[30] Bertlich M Ihler F Weiss BG Freytag S Strupp M Canis M . Cochlear pericytes are capable of reversibly decreasing capillary diameter in vivo after tumor necrosis factor exposure. Otol Neurotol. 2017;38(10):e545-e550. doi:10.1097/MAO.0000000000001523 29135875

[31] Ihler F Sharaf K Bertlich M , et al. Etanercept prevents decrease of cochlear blood flow dose-dependently caused by tumor necrosis factor alpha. Ann Otol Rhinol Laryngol. 2013;122(7):468-473. doi:10.1177/000348941312200711 23951701

[32] Canis M Arpornchayanon W Messmer C Suckfuell M Olzowy B Strieth S . An animal model for the analysis of cochlear blood flood disturbance and hearing threshold in vivo. Eur Arch Otorhinolaryngol. 2010;267(2):197-203. 19597836 10.1007/s00405-009-1036-2

[33] Russel W Burch L . The Principles of Humane Experimental Technique, special ed. Universities Federation for Animal Welfare; 1992.

[34] Klyscz T Jünger M Jung F Zeintl H . [Cap image—a new kind of computer-assisted video image analysis system for dynamic capillary microscopy]. Biomed Tech Biomed Eng. 1997;42(6):168-175. 10.1515/bmte.1997.42.6.168 9312307

[35] Baker M Wayland H . On-line volume flow rate and velocity profile measurement for blood in microvessels. Microvasc Res. 1974;7(1):131-143. doi:10.1016/0026-2862(74)90043-0 4821168

[36] Hibino H Nin F Tsuzuki C Kurachi Y . How is the highly positive endocochlear potential formed? The specific architecture of the stria vascularis and the roles of the ion-transport apparatus. Pflügers Arch. 2010;459(4):521-533. doi:10.1007/s00424-009-0754-z 20012478

[37] Foster CA Breeze RE . The Meniere attack: an ischemia/reperfusion disorder of inner ear sensory tissues. Med Hypotheses. 2013;81(6):1108-1115. 24199949 10.1016/j.mehy.2013.10.015

[38] Lamm K Arnold W . The effect of blood flow promoting drugs on cochlear blood flow, perilymphatic pO2 and auditory function in the normal and noise-damaged hypoxic and ischemic guinea pig inner ear. Hear Res. 2000;141(1-2):199-219. doi:10.1016/s0378-5955(00)00005-8 10713508

[39] Wimbiscus M Kostenko O Malone D . MAO inhibitors: risks, benefits, and lore. Cleve Clin J Med. 2010;77(12):859-882. doi:10.3949/ccjm.77a.09103 21147941

[40] Gaweska H Fitzpatrick PF . Structures and mechanism of the monoamine oxidase family. Biomol Concepts. 2011;2(5):365-377. doi:10.1515/BMC.2011.030 22022344 PMC3197729

[41] Matos MJ Rodríguez-Enríquez F Borges F , et al. 3-amidocoumarins as potential multifunctional agents against neurodegenerative diseases. ChemMedChem. 2015;10(12):2071-2079. 26493007 10.1002/cmdc.201500408

[42] Abdanipour A Jafari Anarkooli I Shokri S Ghorbanlou M Bayati V Nejatbakhsh R . Neuroprotective effects of selegiline on rat neural stem cells treated with hydrogen peroxide. Biomed Rep. 2018;8(1):41-46. 29399337 10.3892/br.2017.1023 PMC5772055

[43] Cui Y Liu KW Liang Y Ip MS Mak JC . Inhibition of monoamine oxidase-B by selegiline reduces cigarette smoke-induced oxidative stress and inflammation in airway epithelial cells. Toxicol Lett. 2017;268:44-50. 28108387 10.1016/j.toxlet.2017.01.005

[44] Claes J Van De Heyning PH . Medical treatment of Meniere’s disease: a review of literature. Acta Otolaryngol. 1997;117(sup526):37-42. 10.3109/00016489709124019 9107354

[45] Ramos Alcocer R Ledezma Rodríguez JG Navas Romero A , et al. Use of betahistine in the treatment of peripheral vertigo. Acta Otolaryngol. 2015;135(12):1205-1211. 26245698 10.3109/00016489.2015.1072873

[46] Marconi S Zwingers T . Comparative efficacy of selegiline versus rasagiline in the treatment of early Parkinson’s disease. Eur Rev Med Pharmacol Sci. 2014;18(13):1879-1882. 25010617

[47] Cereda E Cilia R Canesi M , et al. Erratum to: Efficacy of rasagiline and selegiline in Parkinson’s disease: a head-to-head 3-year retrospective case-control study. J Neurol. 2017;264(9):2051. 28831499 10.1007/s00415-017-8585-x PMC5587620

[48] Müller T Hoffmann JA Dimpfel W Oehlwein C . Switch from selegiline to rasagiline is beneficial in patients with Parkinson’s disease. J Neural Transm. 2013;120(5):761-765. 23196982 10.1007/s00702-012-0927-3

